# Short- and Long-Term Effectiveness of a Subject’s Specific Novel Brain and Vestibular Rehabilitation Treatment Modality in Combat Veterans Suffering from PTSD

**DOI:** 10.3389/fpubh.2015.00151

**Published:** 2015-06-01

**Authors:** Frederick Robert Carrick, Guido Pagnacco, Kate McLellan, Ross Solis, Jacob Shores, Andre Fredieu, Joel Brandon Brock, Cagan Randall, Cameron Wright, Elena Oggero

**Affiliations:** ^1^Carrick Institute, Cape Canaveral, FL, USA; ^2^Global Clinical Scholars Research Training Program, Harvard Medical School, Boston, MA, USA; ^3^Neurology, Carrick Brain Center, Dallas, TX, USA; ^4^Electrical and Computer Engineering, University of Wyoming, Laramie, WY, USA

**Keywords:** PTSD, vestibular rehabilitation, brain rehabilitation, off vertical axis rotation, DSM-IV CAPS

## Abstract

**Introduction:**

Treatment for post-traumatic stress disorder (PTSD) in combat veterans that have a long-term positive clinical effect has the potential to modify the treatment of PTSD. This outcome may result in changed and saved lives of our service personnel and their families. In a previous before–after-intervention study, we demonstrated high statistical and substantively significant short-term changes in the Clinician Administered DSM-IV PTSD Scale (CAPS) scores after a 2-week trial of a subject’s particular novel brain and vestibular rehabilitation (VR) program. The long-term maintenance of PTSD severity reduction was the subject of this study.

**Material and methods:**

We studied the short- and long-term effectiveness of a subject’s particular novel brain and VR treatment of PTSD in subjects who had suffered combat-related traumatic brain injuries in terms of PTSD symptom reduction. The trial was registered as ClinicalTrials.gov Identifier: NCT02003352. We analyzed the difference in the CAPS scores pre- and post-treatment (1 week and 3 months) using our subjects as their matched controls.

**Results:**

The generalized least squares (GLS) technique demonstrated that with our 26 subjects in the 3 timed groups the *R*^2^ within groups was 0.000, *R*^2^ between groups was 0.000, and overall the *R*^2^ was 0.000. The GLS regression was strongly statistically significant *z* = 21.29, *p* < 0.001, 95% CI [58.7, 70.63]. The linear predictive margins over time demonstrated strong statistical and substantive significance of decreasing PTSD severity scores for all timed CAPS tests.

**Discussion:**

Our investigation has the promise of the development of superior outcomes of treatments in this area that will benefit a global society. The length of the treatment intervention involved (2 weeks) is less that other currently available treatments and has profound implications for cost, duration of disability, and outcomes in the treatment of PTSD in combat veterans.

## Introduction

Treatment for post-traumatic stress disorder (PTSD) in combat veterans that have a long-term positive clinical effect has the potential to modify the treatment of PTSD. This outcome may result in changed and saved lives of our service personnel and their families. In a previous before–after-intervention study (1), we demonstrated strong statistical and substantively significant short-term changes of the Clinician Administered DSM-IV PTSD Scale (CAPS) scores (2) in a cohort of 98 combat veterans after a 2-week trial of a subject’s specific novel brain and vestibular rehabilitation (VR) program. The CAPS is considered to be the gold standard for diagnosing PTSD and assessing symptom severity (2, 3). Our sample was gathered from around the United States and we noted a limitation of our study specific to the gathering of long-term post-treatment CAPS scores. The positive changes observed 1 week post-treatment resulted in increased activities of daily living including gainful employment such that our subjects were not able to take the time to travel to our facility for long-term (3-month post-treatment) testing. We therefore decided to repeat our study with a cohort of combat veterans with PTSD gathered from our local clinical area in Dallas, TX, USA in order to facilitate the long-term outcomes testing. Combat veterans residing near our clinic would not have the need for long distance travel and time away from work and lifestyle. Mild traumatic brain injury (mTBI) is the most prevalent injury (4) suffered by combat troops in Iraq/Afghanistan and is significantly associated with blast-related head injuries (5). In fact, service personnel that have suffered mTBI have an increase in PTSD symptoms (6, 7) and there is an elevated prevalence of both PTSD and mTBI among combat veterans (8–10). We know that PTSD and mTBI symptoms are interdependent and mutually influence one another such that a reduction in PTSD symptoms is positively associated with a reduction in post concussive symptoms (11). However, vestibular complaints are the most frequent sequelae of mTBI with VR established as the best treatment modality (12, 13) that is central to our subject’s specific brain and VR rehabilitation of PTSD (1). The PTSD Guideline Development Group and the National Collaborating Centre for Mental Health review team developed a guideline (NICE) based on best available evidence to advise on the treatment and management of PTSD (14). NICE recommended three treatments (Selective serotonin re-uptake inhibitors (SSRI), Eye movement desensitization and reprocessing (EMDR) and trauma-focused cognitive behavioral therapy (TF-CBT). A meta-analysis revealed that TF-CBT is most effective of the three NICE recommended treatments and was associated with the best outcome (15–17). However, immediate post-treatment CAPS outcomes after a 2-week subject’s specific brain and VR treatment program of PTSD after mTBI (1) are associated with strong substantive and statistically significant decreases in PTSD severity. The intervention that we investigated appears to be superior to the NICE recommended treatments. The long-term maintenance of PTSD severity reduction was the subject of this study.

## Materials and Methods

### Research aims and hypothesis

#### Research Question

What is the long-term effectiveness in terms of PTSD symptom reduction of a 2-week subject’s specific novel brain and VR treatment intervention in patients with PTSD who have suffered combat-related traumatic brain injuries.

#### Hypothesis

A 2-week subject’s specific novel brain and VR treatment program will be effective in PTSD symptom reduction shortly after treatment and over time (1 week and 3 months post-treatment).

### Preliminary data

We searched a variety of databases for randomized controlled trials of mTBI and PTSD and VR up until January 2015 without success. Our search included Cochrane Injuries Group’s specialized register, Cochrane Depression, Anxiety and Neurosis Group’s specialized register, Cochrane Central Register of Controlled Trials, MEDLINE, PsycINFO, EMBASE, CINAHL, AMED, ERIC, and PsycBITE. Our team has reported that a subject’s specific novel a 2-week brain and VR treatment has resulted in strong statistical and substantive significance in decreasing PTSD severity in combat veterans (1) as measured by changes in CAPS DSM-IV scores. We reported difficulty in obtaining long-term (3 months) CAPS DSM-IV outcome data due to geographical constraints and the inability of subjects to return for testing associated with increased work and social activities. We addressed these difficulties and proposed a new study involving combat veterans with PTSD who lived in the area of our Institutional Brain Center. The 2008 Institute of Medicine review of interventions research for PTSD concluded that new, well-designed studies are needed to evaluate the efficacy of treatments for PTSD (18). We had completed an initial pilot project with great success and had the funding and patient population to design and implement a long-term study. TBI may reflect an overlap between brain regions vulnerable to traumatic brain injury, and the neural circuitry of these disorders (19).

### Research design and methods

The study was approved by our Institutional IRB and conducted in accordance with the principles of the Declaration of Helsinki. The trial was registered with a service of the U.S. National Institutes of Health as ClinicalTrials.gov Identifier: NCT02003352. There was equipoise.

### Study design

This before–after short- and long-term intervention trial was designed to identify the effectiveness of a subject’s specific novel brain and VR treatment modality in patients with PTSD who have suffered combat-related traumatic brain injuries. Subjects served as their own matched controls. We accomplished the specific aim of our study by analyzing the difference in the CAPS DSM-IV scores (2) pre and post 1 week and 3 months treatment in our subjects as outcomes to compare the effectiveness of the interventions. We expected immediate positive changes in the outcomes after treatment, confirming the results of our previous study, as well as at follow-up over time, i.e., 3 months after cessation of the 2-week treatment period. One qualified licensed psychologist who was blinded to all components of the study conducted all CAPS testing. The study design included one pre-treatment assessment and two post-treatment assessments (at 1 week and 3 months). The study was performed at the Department of Neurology of the Carrick Brain Center in Dallas, TX, USA.

### Sample size

**Figure 1:**
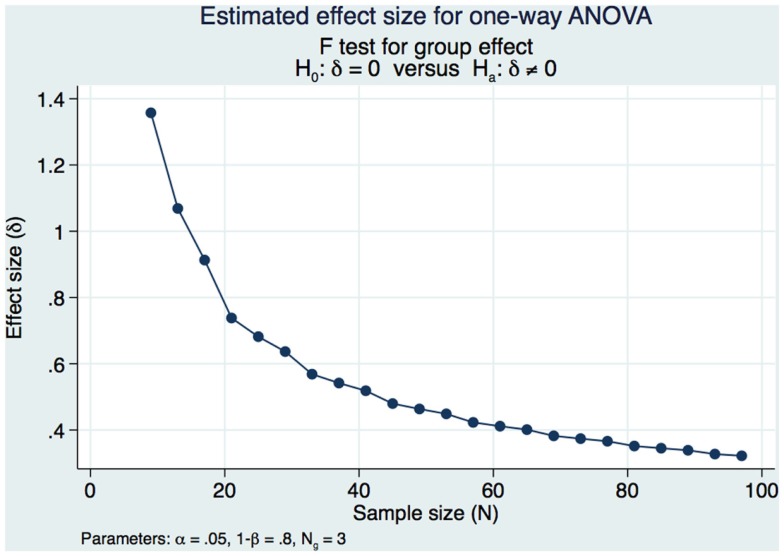
**Estimated effect size for one-way ANOVA**.

Our sample size calculations were based on obtaining a moderately strong estimated effect size (ES) of differences in DSM-IV CAPS scores over time (pre-treatment, 1 week post-treatment and 3 months post-treatment). Our pilot study (1) demonstrated strong statistical and substantive significance of changes in CAPS DSM-IV severity scores in a cohort of 98 subjects that had a pre-treatment mean of 73.13 and a 1-week post-treatment mean of 58.14 (SD of the differences of 14.49 maintaining an α of 0.05 and a power of 80% with subjects serving as their own matched control). We would need 10 subjects serving as their own matched control in the present study if we were to address only the pre-treatment and 1 week post-treatment if our results were as strong as those of our pilot study. However, this present study sample and power calculation is specific to obtaining a moderately strong ES between the pre and 1-week post-treatment CAPS DSM-IV and the long-term 3-month outcomes. We had demonstrated strong statistical and substantive significance in our pilot study and decided that we would accept a long-term ES that was moderately strong, expecting to see a decrease in the effect of the treatment over time while still demonstrating significant statistical and substantive significance from the pre-treatment outcomes. We used Cohen’s *f* (20) to measure the ES and calculate the power and sample size in this study. Cohen’s *f* is the square root of the ratio of the ANOVA between-group to the within-group variances with an *f* = 0.10 defined as a small ES, an *f* = 0.25 is defined as a medium ES, and an *f* = 0.40 is defined as a large ES. We needed 3 groups of 26 subjects for a total of 78 subjects to obtain a moderately strong ES of 0.3586 for a one-way ANOVA. This study design maintained a Type I error at an acceptable level of 0.05 in order to minimize the risk of false positive findings with 80% power (Figure 1).

### Participants

The study population consisted of 26 combat veterans who had suffered a traumatic brain injury with PTSD and were referred to our study by Veteran’s groups. All subjects were male with a mean age of 38.54 years with a minimum age of 25 and a maximum age of 58. They served as their own matched controls and composed three groups of timed diagnostic testing (pre-treatment, 1-week post-treatment, and 3-month post-treatment). They all met the inclusion requirements and did not have any of the exclusion requirements.

### Inclusion criteria

Military combat veterans who had suffered TBI and PTSD that were exposed to war-zone events in Operation Enduring Freedom (OEF) and/or Operation Iraqi Freedom (OIF). Subjects fulfilled all criteria for a diagnosis of chronic PTSD based on the DSM-IV (21) with qualifying scores on the Clinician Administered PTSD Scale (CAPS) (2). All subjects must have had previous treatments for PTSD that was not successful. Subjects were 18 years of age or older and were able to give written informed consent.

### Exclusion criteria

Presence of any of the following DSM-IV diagnoses: psychotic disorder, mania, or bipolar disorder; current major depression with psychotic features; current drug or alcohol or substance/drug dependence; patients that are considered a suicidal risk.

### Assessments and outcome measures

We used changes in the DSM-IV CAPS scores before and after treatment (1 week and 3 months) to distinguish between the estimated frequency and intensity of the various symptoms. Frequency and intensity scores were combined to give a total CAPS score (range: 0–136) as standard in the DSM-IV CAPS evaluation procedures (2, 3). CAPS testing was scheduled pre-intervention, 1 week post-intervention, and at 3 months post-intervention.

### Intervention: Subject’s specific brain and vestibular rehabilitation

We utilized the same subject’s specific novel brain and VR that we reported in our pilot study (1). Our treatments are based upon a combination of VR techniques that include combinations of active and passive head movements in a variety of planes while the subject maintained visual fixation on a target, off axis whole body rotation, visual pursuit, and visual saccadic eye movements to novel targets that are individualized and specific to each subject (22–26). Although the treatment parameters are similar, they are uniquely tailored from subject to subject. For example, one subject might have a deficit of gaze holding in right gaze and another in left gaze. Both would have gaze holding strategies prescribed specific to their clinical needs, the first subject to the right, and the second to the left. To avoid inter-rater variability, the same clinician decided the treatment plan for all subjects. Our clinical experience has shown that customized treatment based on reported symptoms and finding of physical and neurological examination by trained clinicians is more effective than standard VR treatment. Each subject received three daily sessions of VR treatment modalities for 2 weeks (five week days per week with two weekend days off). Subjects were instructed to rest between treatments. No medication changes were prescribed during the treatment period. Clinicians certified in VR administered the treatment. These clinicians did not know the results of the CAPS pre-treatment evaluation.

### Procedure

All subjects met the inclusion criteria at the referring agency and then underwent a comprehensive medical history and neurological examination as well as a CAPS test to confirm PTSD after mTBI. They were carefully examined to ensure that they did not meet any of the exclusion criteria. The subjects that were acceptable to the study were given a detailed explanation of the study and an offer to participate in the study after giving informed consent. Participants underwent another CAPS test 1 week after their treatment had been finished and were scheduled again at 3 months post-treatment.

### Statistical analyses

A statistical analysis was conducted with Stata/SE 13.1 (StataCorp, College Station, TX, USA) according to an intention-to-treat (ITT) approach. Since it was expected that some participants might not complete the study for a variety of reasons, the analysis procedure included provisions for identifying these individuals making a careful note of the reasons for no completion if possible. However, compliance with treatment appointments was necessary for the subject to be included in the analysis. An individual who missed more than 25% of their treatment appointments would be categorized as being non-compliant. Dropouts were to be identified separately from those individuals who were deemed non-compliant. The ANCOVA served as our design substitute for randomization. The short-term efficacy of the treatment modality was evaluated by considering the difference pre and post (at 1 week after treatment) of the CAPS Total Severity Scores for each subject (matched pairs) and by calculating the probability of error (*p* value) by a two-tailed *t*-test for repeated measures maintaining an α of <0.05. The ES was calculated in several ways to ascertain if the difference between the matched pairs was both statistically and clinically significant: as proposed by Cohen, the ratio of the mean difference between the two groups (pre and 1 week post) divided by the pooled variance of the groups was calculated (27). Another ES as proposed by Hedges was calculated using a formula similar to Cohen’s but calculating the SD using *N*−1 instead of *N* (where *N* is the number of samples considered) (28). In both cases, an ES value of 0.2 represents a small statistical and clinical difference between two groups; an ES value of 0.5 represents a moderate difference; and an ES value of 0.8 represents a large difference (27). The ES was also obtained by calculating the point biserial correlation: a percent improvement between CAPS Total Severity Score was calculated [(post test group mean minus pre test group mean)/(pre test group mean) × 100], the changes between sequential CAPS scores were measured and how strong the relationship was between them was calculated. In this last case, a value of 0.01–0.09 is a small effect, a value of 0.10–0.25 is a medium effect and a value of over 0.25 represents a large ES. Similar calculations were done to assess the long-term efficacy of the treatment modality by considering the difference pre and post (at 3 months after treatment) of the CAPS Total Severity Scores for each subject (matched pairs). We also used a multiple linear regression model to predict and explain both the short- (1 week post-treatment) and long-term (3 month) outcomes and included Eta Squared (η^2^) and Omega Squared (ω^2^) calculations of the ES. We calculated the Eta Squared (η^2^) by taking the sum of squares for a variable divided by the total sum of squares to reveal how much of the variation in the sample was explained by the predictor. Cohen (29) suggests that a value of η^2^ of 0.01 is a small ES, 0.06 is medium, and 0.14 is large. The ω^2^ is an estimate of the explained variable in the population and adjusts for degrees of freedom and the error term, making it somewhat smaller than the η^2^. We also calculated the Beta Weights (β) as a measure of the ES of the multiple linear regression. β = 0.01 is a weak effect, β = 0.30 is a moderate effect, and β = 0.50 is a strong effect. Furthermore, we calculated the correlation between our timed tests (a correlation of |*r*| = 0.1 is a weak relationship, |*r*| = 0.3 is a moderate relationship, and |*r*| = 0.5 is a strong relationship). Finally, we used a repeated measure ANOVA to evaluate the three groups over time in this before–after-intervention study to ascertain if the intervention was enduring as well as ANCOVA testing as an extension of our multiple regression model. We desired to control for group differences that might influence the results because of our lack of ability to randomize our sample. Residuals were analyzed for normalcy and compared with predicted values to discern any possible issues with our model. A residual-vs.-fitted plot was developed to see if we could discern a pattern that would indicate that our model has problems, and an Adjusted Partial Residual Plot using regressors already in the model was used to better understand the regression. Correlation between the CAPS scores over time was calculated.

We also decided to use a bootstrap estimation of the SEs in the linear regression. We drew 1000 random samples with replacement from the dataset and examined the distribution of each parameter using the variance of that distribution to estimate a SE.

Finally, we wanted to estimate any unknown parameters in our linear regression model of our three treatment groups by using a random effects generalized least squares (GLS) technique and by doing an ANOVA of the changes in CAPS scores over time.

## Results

**Figure 2:**
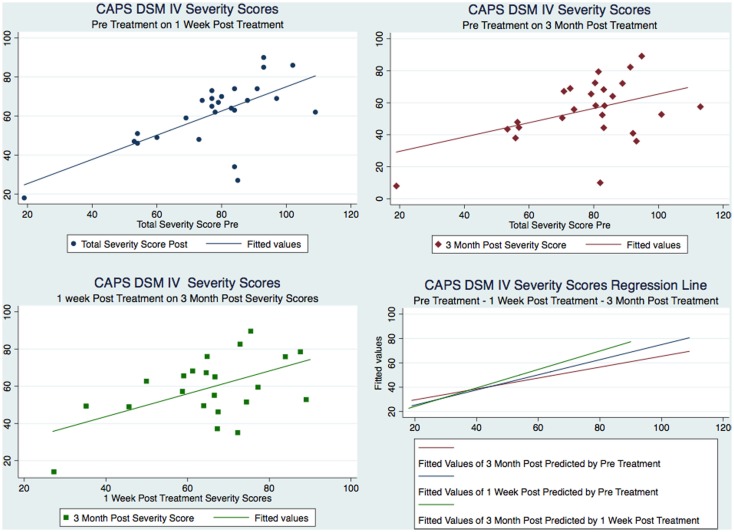
**Figure 2 CAPS severity scores for each of the paired observations and the regression line fitting the values**.

Our sample was composed of 26 males with a mean age of 38 that served as their own matched controls in three groups representing CAPS DSM-IV testing immediately pre-treatment (mean 77.58, SD 18.45), 1 week post-treatment (mean 61.08, SD 17.29), and at 3 months post-treatment (mean 55.38, SD 18.43). Figure 2 shows the CAPS severity scores for each of the paired observations Pre – 1-week post; Pre – 3-month post; 1-week post – 3-month post, for all the subjects as well as the regression line fitting the values. As it is evident from the plot of the three superimposed fitted lines, the regression line of the 1-week post – 3-month post pair has the greater slope, whereas the one of the Pre – 3-month post pair has the lower slope.

**Figure 3:**
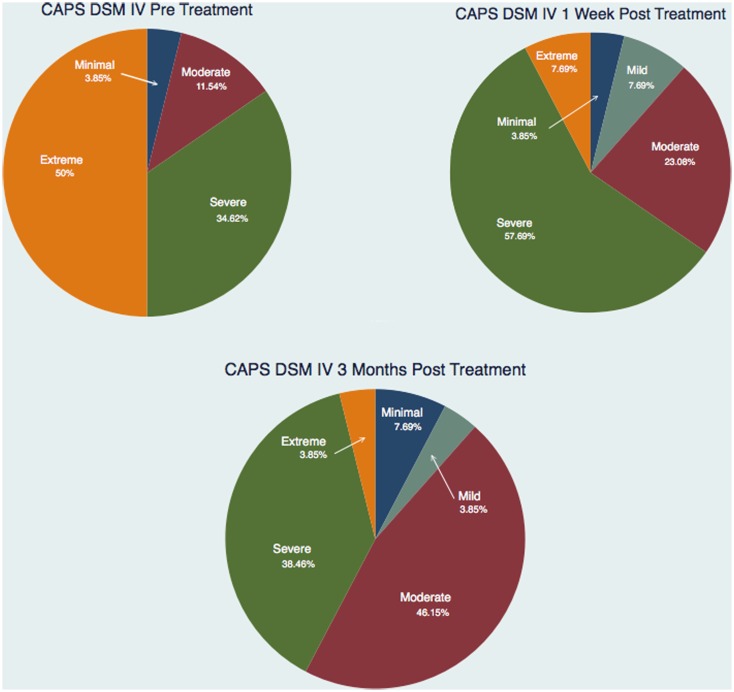
**Number of subjects for each of the categories of the CAPS DSM IV scores for each of the three testing sessions**.

Figure 3 and Tables 1–3 show the number of subjects for each of the categories of the CAPS DSM-IV scores (minimal, mild, moderate, severe, extreme) for each of the three testing sessions (pre-treatment, 1-week post-treatment, and 3-month post-treatment). It is evident that the treatment is effective and the number of subjects included in the last category (Extreme) gets significantly smaller over time and there is a distribution of the subjects toward lesser categories (less symptoms).

**Table 1:** **Two way table with measures of association for the CAPS total severity scores pre and 1 week post-treatment divided into each category (minimal, mild, moderate, severe, and extreme) and their relative percentage**.

Severity category pre	Severity category post					
	Minimal	Mild	Moderate	Severe	Extreme	Total
Minimal 0–19	1	0	0	0	0	1
	100	0.00	0.00	0.00	0.00	100.00
	100	0.00	0.00	0.00	0.00	3.85
Mild 20–39	0	0	0	0	0	0
	0.00	0.00	0.00	0.00	0.00	0.00
	0.00	0.00	0.00	0.00	0.00	0.00
Moderate 40–59	0	0	3	0	0	3
	0.00	0.00	100.00	0.00	0.00	100.00
	0.00	0.00	50.00	0.00	0.00	11.54
Severe 60–79	0	0	3	6	0	9
	0.00	0.00	33.33	66.67	0.00	100.00
	0.00	0.00	50.00	40.00	0.00	34.62
Extreme 80–136	0	2	0	9	2	13
	0.00	15.38	0.00	69.23	15.38	100.00
	0.00	100	0.00	60.00	100.00	50.00
Total	1	2	6	15	2	26
	3.85	7.69	23.08	57.69	7.69	100.00
	100.00	100.00	100.00	100.00	100.00	100.00

*χ^2^(12) = 43.0667, *p* = 0.000, ϕ = 0.7431*.

*Key: frequency; row percentage; column percentage*.

**Table 2:** **Two way table with measures of association for the CAPS total severity scores pre and 3 month post-treatment divided into each category (minimal, mild, moderate, severe, and extreme) and their relative percentage**.

Severity category pre	Severity category post					
	Minimal	Mild	Moderate	Severe	Extreme	Total
Minimal 0–19	1	0	0	0	0	1
	100	0.00	0.00	0.00	0.00	100.00
	50	0.00	0.00	0.00	0.00	3.85
Mild 20–39	0	0	0	0	0	0
	0.00	0.00	0.00	0.00	0.00	0.00
	0.00	0.00	0.00	0.00	0.00	0.00
Moderate 40–59	0	0	3	0	0	3
	0.00	0.00	100.00	0.00	0.00	100.00
	0.00	0.00	25.00	0.00	0.00	11.54
Severe 60–79	0	0	4	5	0	9
	0.00	0.00	44.44	55.56	0.00	100.00
	0.00	0.00	33.33	50.00	0.00	34.62
Extreme 80–136	1	1	5	5	1	13
	7.69	7.69	38.46	38.46	7.69	100.00
	50.00	100	41.67	41.67	100.00	50.00
Total	2	1	12	10	1	26
	7.69	3.85	7.69	38.46	3.85	100.00
	100.00	100.00	100.00	100.00	100.00	100.00

*χ^2^(12) = 18.7407, *p* = 0.095, ϕ = 0.4902*.

*Key: frequency; row percentage; column percentage*.

**Table 3:** **Two way table with measures of association for the CAPS total severity scores 1-week post and 3-month post-treatment divided into each category (minimal, mild, moderate, severe, and extreme) and their relative percentage**.

Severity category pre	Severity category post					
	Minimal	Mild	Moderate	Severe	Extreme	Total
Minimal 0–19	1	0	0	0	0	1
	100	0.00	0.00	0.00	0.00	100.00
	50	0.00	0.00	0.00	0.00	3.85
Mild 20–39	1	0	1	0	0	2
	50.00	0.00	50.00	0.00	0.00	100.00
	50.00	0.00	8.33	0.00	0.00	769
Moderate 40–59	0	0	4	2	0	6
	0.00	0.00	66.67	33.33	0.00	100.00
	0.00	0.00	33.33	20.00	0.00	23.08
Severe 60–79	0	1	6	8	0	15
	0.00	6.67	40.00	53.33	0.00	100.00
	0.00	100.00	50.00	80.00	0.00	57.69
Extreme 80–136	0	0	1	0	1	2
	0.00	0.00	50.00	0.00	50.00	100.00
	0.00	0.00	8.33	0.00	100.00	7.69
Total	2	1	12	10	1	26
	7.69	3.85	46.15	38.46	3.85	100.00
	100.00	100.00	100.00	100.00	100.00	100.00

*χ^2^(12) = 34.2044, *p* = 0.095, ϕ = 0.5735*.

*Key: frequency; row percentage; column percentage*.

**Figure 4:**
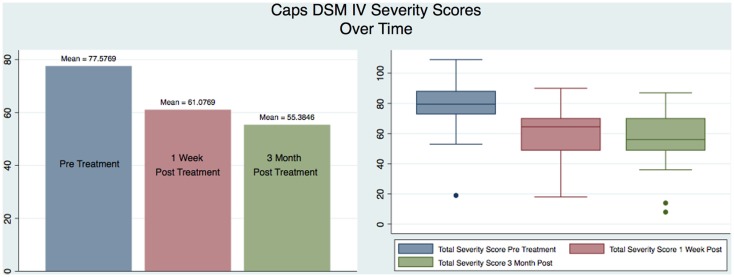
**Caps DSM IV Severity Scores Over Time**.

Figure 4 shows a graphical representation of the statistical values (mean and box plot) for all the subjects for each of the three testing sessions. It is immediately apparent that there is a reduction in the severity of the CAPS scores from the pre to the 1-week post-treatment confirming the results previously obtained (1), but also such reduction continues over time as measured by the 3-month post CAPS scores.

This decrease in symptoms measured as reduction in the CAPS Score is statistically significant: Table 4 includes the *t*-tests results for each comparison (Pre – 1-week post; Pre – 3-month post; 1-week post – 3-month post) for all the subjects as well as for those in the moderate, severe, and extreme categories of the CAPS scores pre-treatment: only the moderate category is not significant, but the results could be heavily affected by having only three subjects in this category. The minimal (one subject pre-treatment) and mild (no subjects pre-treatment) categories were not considered separately given the small or inexistent number of subjects in these categories.

**Table 4:** **Two-tailed paired *t*-tests**.

Groups	Variable 1	Variable 2	*T*	*p*
All subjects (26)	Pre	1-week Post	5.72	0.0000*
	Pre	3-month Post	5.84	0.0000*
	1-week Post	3-month Post	2.13	0.0428*
Pre-treatment extreme (13)	Pre	1-week Post	4.90	0.0004*
	Pre	3-month Post	5.38	0.0002*
	1-week Post	3-month Post	2.10	0.0578
Pre-treatment severe (9)	Pre	1-week Post	5.62	0.0005*
	Pre	3-month Post	4.86	0.0013*
	1-week Post	3-month Post	0.25	0.8052
Pre-treatment moderate (3)	Pre	1-week Post	3.90	0.0599
	Pre	3-month Post	3.47	0.0741
	1-week Post	3-month Post	1.14	0.3715

*The significant values.

Table 5 shows the ESs based on mean comparison for the Pre – 1-week post and the Pre – 3-month post-treatment groups of CAPS scores. For the Cohen’s *d* and Hedges’s *g*, an ES value of 0.2 represents a small statistical and clinical difference between two groups; an ES value of 0.5 represents a moderate difference; and an ES value of 0.8 represents a large difference (27), and for the Point Biserial *r* a value of 0.01–0.09 is a small effect, a value of 0.10–0.25 is a medium effect, and a value of over 0.25 represents a large ES. Therefore, all three methods show a large difference or effect, making the difference not only statistically, but also substantively significant.

**Table 5:** **Effect size based on mean comparison**.

Groups	Variable 1	Variable 2	Estimate	95% CI
Cohen’s *d*	Pre	1-week Post	0.92	0.35–1.49
	Pre	3-month Post	1.20	0.61–1.79
Hedges’s *g*	Pre	1-week Post	0.91	0.34–1.47
	Pre	3-month Post	1.19	0.60–1.76
Point Biserial *r*	Pre	1-week Post	0.43	0.17–0.61
	Pre	3-month Post	0.29	0.30–0.67

**Figure 5:**
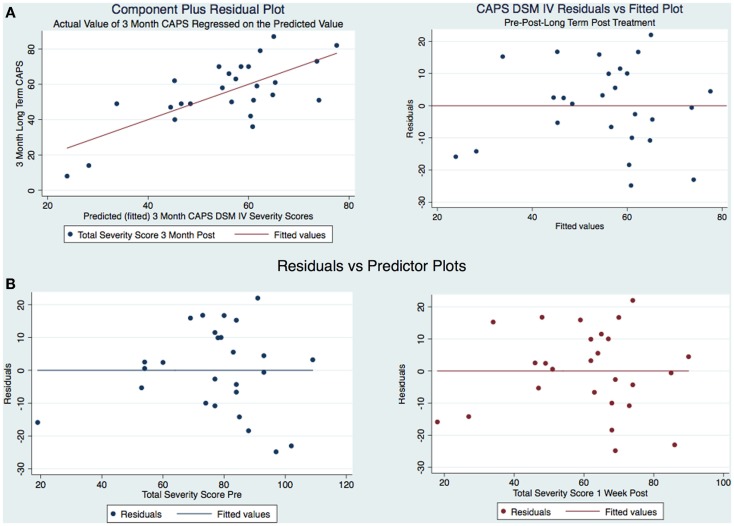
**(A)** CAPS DSM-IV component-plus-residual plot and residuals vs. fitted and **(B)** Residual vs. predicted plots.

Our sample was not randomized and we used an analysis of covariance (ANCOVA) and a multiple linear regression model to statistically control for group differences that might influence the result because we could not rule out these possible differences through randomization. There was a strong statistically significant difference between the means [*F* = (2,23) = 11.85 *p* < 0.001]. The ANCOVA and linear regression model predicted 51% of the model (*R*^2^ = 0.5075, Adjusted *R*^2^ = 0.46). The SD around the regression line is much smaller than the SD around the mean [root mean squared error (MSE) = 13.49] and improves our prediction. The regression equation allows us to estimate the 3-month CAPS scores depending on the pre-treatment and 1-week post-treatment CAPS scores. A 1 point increase in the pre-treatment CAPS score is associated with a decrease in the 3-month CAPS score of −0.04; however, this is not statistically significant [*t*(23) = 0.83, *p* = 0.83]. The 95% confidence interval has a range from −0.45 to 0.36 and contains a 0 signifying no statistically significant relationship. An increase in the CAPS score of 1 point on the 1-week post CAPS scores is associated with a decrease in the 3-month CAPS score of 0.79 points with strong statistical significance (*t*(23) = 3.79, *p* < 0.001, CI [0.3577556, 1.219584]). We are 95% confident that the interval of 0.36–1.21 contains the true slope. We also calculated Beta (β) weights as a measure of the ES and found that they were substantively significant with the pre-treatment β = −0.042 (moderate) and the 1-week post-treatment β = 0.74 (extremely strong). We wanted to see if the residuals were normally distributed. Therefore, it appears that the CAPS score 1-week post-treatment better correlates with the CAPS scores 3-month post-treatment than the CAPS score pre-treatment. This has important clinical consequences: the CAPS score pre-treatment is not an indication of how the subject will respond to the subject specific brain and VR therapy. However, as soon as the 2-week treatment regimen is completed and the subject is evaluated 1-week post-treatment, if there has been a change in the CAPS score over such a short time, it is a good indication that not only the subject will maintain such improvement but also that improvement will be continuing over the next 3 months. The skewness of the residuals is −0.23 compared to the value of 0.00 for a normal distribution. The kurtosis is 2.16 compared with the value of 3.00 for a normal distribution. Both the skewness and kurtosis of the residuals were not significantly different from what they would be if our residuals were normally distributed. *p* = 0.58 for Skewness and *p* = 0.33 for Kurtosis. This was done because we wanted to look at the distribution of residuals for the different predicted values to see if the residuals were distributed similarly to the predicted values. Therefore, after having verified that the residuals could be considered normally distributed, the residual-vs.-fitted plot was developed (Figure 5).

**Figure 6:**
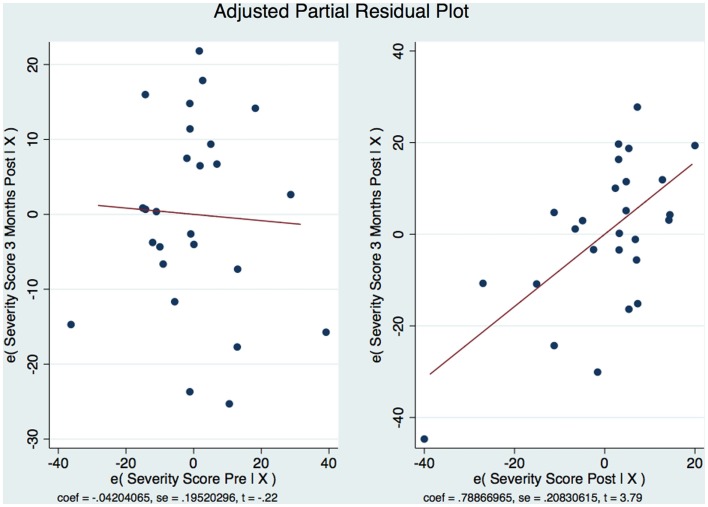
**Adjusted partial residual plot**.

The model is well-fitted and there is no pattern to the residuals plotted against the fitted values with no violation of the least-squares assumptions. Note that where the fitted values are low between 20 and 30, the residuals tend to be negative and where the fitted values are moderate between 30 and 50 the residuals tend to be positive. The fitted values over 50 are associated with residuals that tend to be similar to the predicted values with the observations normally distributed for any fitted values (*x* axis) about the reference line (residual of 0 on *y* axis). We wanted to see how the residuals are distributed by graphing the actual 3-month CAPS DSM-IV score on a predicted value for this score. Using the component-plus-residual plot to assist in projecting multidimensional data into a two-dimensional form (Figure 5A), we can examine the functional form assumptions of the model. The regression line through the coordinates has a slope equal to the estimated coefficient in the regression model. By looking at the residuals vs. predictor plots no specific patterns comes to mind, indicating that the model considered takes into account most of the phenomenon and the residuals are indeed random. We then developed an Adjusted Partial Residual Plot using regressors already in the model to better understand the regression (Figure 6). The regression of *y* on *x* has the same coefficient and SE (up to a degree-of-freedom adjustment) as the estimated coefficient and SE for the regressor in the original regression. We are confidant that the residuals are normally distributed and that our model is valid. The pre-treatment CAPS scores were strongly correlated with the 1-week post-treatment CAPS scores (0.66, *p* < 0.001) and moderately correlated with the 3-month post-treatment CAPS scores (0.45, *p* < 0.05). The 1-week post-treatment CAPS scores were strongly correlated with the 3-month post-treatment CAPS scores (0.71), *p* < 0.001.

We decided to use a bootstrap estimation of the SEs in linear regression. We drew 1000 random samples with replacement from the dataset and examined the distribution of each parameter using the variance of that distribution to estimate a SE. The bootstrap estimation did not demonstrate statistical significance of the pre-treatment CAPS as a predictor of the 3-month post-treatment CAPS (*z* = −0.18, *p* = 0.86, 95% CI [−0.51, 0.42]). However, it did demonstrate statistical significance of the 1-week post-treatment CAPS as a predictor of the 3-month post-treatment CAPS scores (*z* = 3.38, *p* < 0.001, 95% CI [0.33, 1.25]). This confirmed what was found in Figure 2. We wanted to estimate any unknown parameters in our linear regression model of our 3 treatment groups by using a random effects GLS technique. The GLS demonstrated that with our 26 subjects in the 3 timed groups the *R*^2^ within groups was 0.000, *R*^2^ between groups was 0.000, and overall the *R*^2^ was 0.000. The GLS regression was strongly statistically significant *z* = 21.29, *p* < 0.001, 95% CI [58.7, 70.63]. An ANOVA of the CAPS tests over time predicted 79% of the variation in the 3-month post-treatment CAPS scores *R*^2^ = 0.79, adjusted *R*^2^ = 0.68. There was a statistically significant relationship between the pre and 1-week post-treatment and the 3-month post-treatment CAPS [*F*(1,77) = 7.12, *p* < 0.001]. We calculated the *R*^2^ for the timed CAPS scores independent of the model (*F* = 26.67, *p* < 0.0001) and adjusted for the potential bias in the *F* statistic by calculating the Huynh–Feldt ε = 0.8800, Greenhouse–Geisser ε = 0.8285, and Box’s conservative ε = 0.5000. These adjustments revealed that all three of the adjustments revealed an *F* of 26.67 with a strong statistical significance of <0.001. The omega squared ES of the timed CAPS tests = 0.6794 representing extremely strong substantive significance. The linear predictive margins over time demonstrated strong statistical significance for all timed CAPS tests. The pre-treatment CAPS *t*(78) = 34.76, *p* < 0.001, 95% CI [73.09, 82.06], 1-week post-treatment CAPS *t*(78) = 27.37, *p* < 0.001, 95% CI [56.59, 65.56], and the 3-month post-treatment CAPS *t*(78) = 24.82, *p* < 0.001, 95% CI [50.90, 59.87] (Table 6).

**Table 6:** Linear predictive margins of CAPS over time with significance*.

Time	Margin	SE	*t*	*p*	95% CI
Pre-treatment	77.58	2.23	34.76	0.000*	73.09–82.06
1-week Post-treatment	61.08	2.23	27.37	0.000*	56.59–65.56
3 Months Post-treatment	55.38	2.23	24.82	0.000*	50.90–59.87

*GLS regression *Z* = 21.29, *p* < 0.001, 95% CI [58.40–70.63]*.

**ω*^2^ effect size of the timed CAPS = 0.6794 (extremely strong substantive significance)*.

## Discussion

We accomplished the specific aim of our study by analyzing the difference in the pre-treatment, 1-week and 3-month post-treatment CAPS severity scores in our subjects as outcomes to compare the effectiveness of the subject’s specific novel brain and VR interventions. We expected to find both statistical and substantive significant decreases in CAPS severity scores similar to our pilot study and we did. However, we expected to see a drop of the treatment effect at 3 months after treatment but found that the subjects continued to improve. We attribute this to the increased brain activation resulting from an increased ability to function in activities of daily living. Our subjects were able to embrace activities, both cognitively and physically that they were not previously able to navigate due to the severity of their PTSD symptoms. Increased activity because of decreased suffering has continued to be associated with a further diminution of PTSD severity scores.

This investigation has analyzed the use of a subject’s specific novel brain and VR treatment modality in PTSD patients who have suffered combat-related traumatic brain injuries immediately and over time after treatment. In general, we obtained both strong statistical and substantive significant outcomes. The treatment of this disorder as a physical injury with brain and vestibular non-invasive and non-pharmaceutical applications over a 2-week program has lasting positive effects. Further, a successful 2-week treatment period may be associated with significant savings of cost, time, and disability when compared to longer therapy programs. There may be a stigma associated with having a neuropsychiatric diagnosis of PTSD that might be lessened if a physicality of etiology similar to that commonly accepted in mTBI is embraced. Our investigation has the promise of development of superior outcomes of treatments in this area that will benefit a global society. The continued decrease of PTSD severity over time is an exciting reality that is uniquely associated with this novel approach to the treatment of a disorder that has personal, familial, and societal consequences. The length of the treatment intervention involved (2 weeks) is less that other currently available treatments and has profound implications on cost, duration of disability, and outcomes in the treatment of PTSD in combat veterans.

### Strengths and weaknesses of the study

The observed clinical results of our treatments might change the direction of therapy sponsored by government and private agencies. We have been successful in obtaining long-term (3 months) outcomes that have answered an important clinical question of treatment effect. This study did not include any female subjects. We are aware that many women are military veterans who have suffered blast injuries and resultant PTSD; however, we wanted to verify the results of our previous study and preferred not to include female subjects to avoid adding another possible confounding factor (the gender). Future research is needed to verify if similar outcomes can be obtained with women.

## Conflict of Interest Statement

The authors declare that the research was conducted in the absence of any commercial or financial relationships that could be construed as a potential conflict of interest.
